# A Highly Sensitive Porous Silicon (P-Si)-Based Human Kallikrein 2 (hK2) Immunoassay Platform toward Accurate Diagnosis of Prostate Cancer

**DOI:** 10.3390/s150511972

**Published:** 2015-05-22

**Authors:** Sang Wook Lee, Kazuo Hosokawa, Soyoun Kim, Ok Chan Jeong, Hans Lilja, Thomas Laurell, Mizuo Maeda

**Affiliations:** 1 Bioengineering Laboratory, RIKEN, Saitama 3510198, Japan; E-Mails: k-hoso@riken.go.jp (K.H.); mizuo@riken.jp (M.M.); 2 Department of Biomedical Engineering, Dongguk University, Seoul 100715, Korea; E-Mail: thomas.laurell@bme.lth.se; 3 Department of Mechanical Engineering, Inje University, Gimhae-si 621-749, Korea; E-Mail: memsoku@inje.ac.kr; 4 Department of Translational Medicine, Lund University, Skåne University Hospital in Malmö, Malmö 20502, Sweden; E-Mail: liljah@mskcc.org; 5 Departments of Laboratory Medicine, Surgery (Urology), and Medicine (GU Oncology), Memorial Sloan-Kettering Cancer Center, New York, NY 10065, USA; 6 Institute for Biosciences and Medical Technology, University of Tampere, Tampere 33800, Finland; 7 Nuffield Department of Surgical Sciences, University of Oxford, Oxford OX3 7DQ, UK; 8 Department Biomedical Engineering, Lund University, Lund 22100, Sweden

**Keywords:** porous silicon, human kallikrein 2, antibody microarrays, prostate cancer

## Abstract

Levels of total human kallikrein 2 (hK2), a protein involved the pathology of prostate cancer (PCa), could be used as a biomarker to aid in the diagnosis of this disease. In this study, we report on a porous silicon antibody immunoassay platform for the detection of serum levels of total hK2. The surface of porous silicon has a 3-dimensional macro- and nanoporous structure, which offers a large binding capacity for capturing probe molecules. The tailored pore size of the porous silicon also allows efficient immobilization of antibodies by surface adsorption, and does not require chemical immobilization. Monoclonal hK2 capture antibody (6B7) was dispensed onto P-Si chip using a piezoelectric dispenser. In total 13 × 13 arrays (169 spots) were spotted on the chip with its single spot volume of 300 pL. For an optimization of capture antibody condition, we firstly performed an immunoassay of the P-Si microarray under a titration series of hK2 in pure buffer (PBS) at three different antibody densities (75, 100 and 145 µg/mL). The best performance of the microarray platform was seen at 100 µg/mL of the capture antibody concentration (LOD was 100 fg/mL). The platform then was subsequently evaluated for a titration series of serum-spiked hK2 samples. The developed platform utilizes only 15 µL of serum per test and the total assay time is about 3 h, including immobilization of the capture antibody. The detection limit of the hK2 assay was 100 fg/mL in PBS buffer and 1 pg/mL in serum with a dynamic range of 10^6^ (10^−4^ to 10^2^ ng/mL).

## 1. Introduction

Human tissue kallikrens (hKs) are secreted from human proteases with diverse expression and physiological roles [1]. They consist of 15 (hK1-hK15) genes located on chromosome 19, in the q13.3–13.4 region. Among them, three kallekreins (hK1, hK2 and hK3) have high amino acid homology each other. hK2 and hK3 (PSA) share 80% amino acids while KLK1 shows 62% to 67% homology with PSA and hK2, respectively [2]. As a biomarker of prostate cancer, the levels of prostate specific antigen (PSA or hK3) in the serum, originating from leakage of pathological tissue to the vascular system, are routinely measured, and have been shown to be proportional to the tumor burden [3]. Even though it is the most commonly used biomarker for diagnosis of prostate cancer, and PSA screening tests are reported to have a significant correlation with 20% reduction in cancer mortality, a PSA concentration above the frequently used diagnosis cutoff (3–4 ng/mL) does not necessarily mean cancer as the serum PSA assay lacks specificity, *i.e.*, the capability of distinguishing prostate cancer from some non-malignant prostatic pathologies such as benign prostatic hyperplasia or prostatitis [4], with the drawback of requiring unnecessary painful biopsies because of the low specificity [5]. It should also be noted that only 25% of men with slightly elevated PSA levels do have PCa [5]. In addition, a PSA concentration below the cutoff may actually be cancer. Moreover there are strong arguments challenging the usefulness of PSA screening tests because of the large discrepancies between decreasing disease aggressiveness and increasing levels of treatment. This has led to widespread criticism that prostate cancer is now an “overdiagnosed” and “overtreated” cancer [6]. Therefore, requirement for additional prostatic tumor markers are increasingly high.

Some other kallikrein family proteins are recognized as useful biomarkers of prostate cancer. It is reported that higher KLK4 mRNA levels in the prostate tissue obtained by biopsy are correlated with prognosis and cancer stage [7]. Kallikrein-related peptidase 5 (KLK5) is overexpressed in normal tissues compared to cancerous prostatic ones and it shows an inverse relationship between KLK5 levels and pathologic tumor stage [8]. Moreover, elevated KLK 11 mRNA expressions have been found to be associated with a less advanced stage, and an optimistic disease course for prostate cancer [7]. Human kallikrein 2 (hK2) is a serine protease with trypsin-like specificity and has many similarities to PSA. Since the expression of hK2 protein is higher in malignant prostatic tissue *versus* benign tissue it has been also considered as a prostate cancer (PCa) biomarker since levels of hK2 in serum from PCa patients are increased relative to individuals with BPH [9,10,11,12]. Expression analysis by RT-PCR has shown the down-regulation of PSA mRNA, while hK2 mRNA is up-regulated in aggressive tumors [13]. It is suggested that hK2 could also be useful in predicting pathologic stage and grade along with biochemical outcome in patients treated with radical prostatectomy [14]. In patients with mildly elevated PSA levels, hK2 acts as an independent predictor for PCa diagnosis [15]. Moreover, hK2 might be suggested as a potential biomarker in diagnosing poorly differentiated tumors [16], as well as differentiating between organ-confined cancer and extra-capsular disease [17].

The concentration of hK2 in human prostates is approximately 10%–50% of the PSA level and it is 50- to 100-fold lower than the PSA concentration in blood serum [18,19]. Despite the intrinsically low expression levels of hK2, development of sensitive assay methods of hK2 is insufficient compared to that of PSA assay [20,21,22,23,24]. Only the Dissociation-Enhanced Lanthanide Fluorescent Immunoassay, the so called DELFIA system that utilizes the unique chemical properties of lanthanide chelates in concert with time-resolved fluorescence (TRF) detection, has reported a limit of detection of hK2 in the low pg/mL with a 10^3^ order (3 pg/mL to 3 ng/mL) dynamic range [10,25]. This method requires laborious labeling procedures and a sophisticated optical system. Therefore, a robust, simple but highly sensitive assay technology for hK2 detection is still required.

The 3-D macro-pore structures of the P-Si enlarges the surface area for immobilization [26,27] and hence results in an increased density of capture antibody on the surface [28]. Since physical adsorption is the main method to bind the antibody on the surface, it provides fast immobilization of capture antibody (after less than a few minutes of incubation) without any chemical treatment on the surface [28]. Antibody or protein (such as IgG and Protein A) is strongly adsorbed on the silicon surface when the molecules are dispensed as small droplets and the droplets are quickly dried out [26,27,28]. Numerous factors, including pore size, protein size, surface chemistry and layer thickness, will influence the amount of protein adsorbed, as well as its structure and function [29,30]. There are hundreds of different cross-linking reagents available, resulting in covalent binding between the biomolecules and silicon surface, however, it is very beneficial to find a surface that adsorbs protein spontaneously since derivation might affect the other surface properties, such as hydrophobicity, fluorescent background and surface charges [30].

P-Si enables one to set up a sensitive and simple assay protocol compared to the other proposed amplification methods such as modifying the detection antibodies, by e.g., dendritic amplification [31], catalyzed signal amplification with colorimetric readout [32,33] or detection with rolling-circle amplification [34]. We previously developed a P-Si (porous silicon) antibody microarray platform for analyzing prostate specific antigen (PSA) in serum [24] and α-synuclein in cerebrospinal fluid (CSF) [35] with high sensitivity and reproducibility. P-Si with sub-micron pores is optimal for antibody immobilization. We investigated immunoassays using a P-Si microarray at three different capture antibody (PSA––prostate specific antigen) concentrations, analyzing the influence of the antibody density on the assay detection sensitivity. The microarray showed a LOD of 800 fg/mL and a dynamic range of 800 fg/mL to 80 ng/mL in serum-spiked PSA [24]. Usage of our P-Si microarray also extends to α-synuclein in CSF which is one of the potential biomarker of Parkinson’s disease. The porous silicon microarray displayed a 35 pg/mL LOD and a dynamic range of four orders of magnitude (17 pg/mL to 500 ng/mL) [35]. The substrate is well suited for surface-based immunoassays because the micro- and nonporous structure of the substrate adsorbs antibodies in an intact state on the enlarged 3-D surface. It can be noted that this surface is also compatible with mass spectrometric readout [28,36]. Table 1 present several supports materials for microarrays and their respective advantages and disadvantages.

**Table 1:** Solid microarray supports.

	Derivatized Glass	Filter/Membrane	Gel Pad/Agarose Film	Porous Silicon
Advantages	Cheap	Low cost	High sample capacity	High sample capacity
	Compatible with most micro-arrayers	Reusable		Spot homogeneity
				Low unspecific binding
Disadvantages	Non-uniform spots (Coffee ring effect)	Limit of spot density	Expensive	Laborious procedures
			Laborious procedures	

The protein/antibody microarray format should ultimately be used not only for qualitative analysis such as profiling the abundance of thousands of proteins [37,38], or globally analyzing protein phosphorylation [39], but also to evolve into the development of a quantitative approach [40,41]. Although the potential of protein microarray technology was foreseen more than 20 years ago by Ekins [42], it is only recently that it started to find its way into proteomics research, diagnosis and drug discovery [43,44,45].

Sandwich immunoassays are widely used for diagnostics, frequently in 96 well formats. They consist of a pair of antibodies (capture antibody and detection antibody) that bind to the target biomarkers in a sandwich format. Sandwich assays offer high detection sensitivity resulting from the enrichment of the target proteins by the capture antibody [46,47].

**Figure 1:**
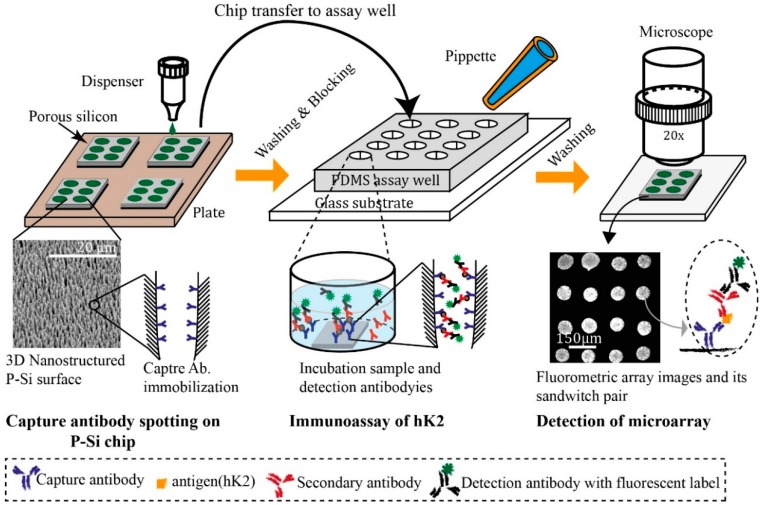
Schematic of the P-Si chip immunoassay procedure, starting with the dispensing of hK2 capture antibodies onto the porous silicon surface. The porous silicon chip with physically adsorbed antibodies is placed into an assay well made of polydimethyl-polysiloxane (PDMS), and hK2-containing serum samples are added; the size of the PDMS assay is well suited for each P-Si chip and makes it easy to perform parallel pipetting. Subsequently, detection antibody (polyclonal primary and Alexa 488 labeled secondary antibody) is used for measuring fluorescent signals under a microscope.

In this study, we developed a P-Si-based antibody microarray platform for improving the detection sensitivity of the hK2 assay. Detection sensitivity and dynamic ranges of the P-Si assay platform were investigated by varying the density of the capture antibody on the spots. Figure 1 shows the procedures of the developed hK2 immunoassay. Three different concentrations of capturing antibody were arrayed on a porous silicon chip and evaluated in the detection hK2 in buffer (PBS). The P-Si antibody microarray format, with optimized capture antibody concentration, was subsequently evaluated in the detection of hK2 spiked into human female serum. We also investigate the cross-reaction between hK2 capture antibodies and PSA antigen since PSA and hK2 antigen have high homology [9,10]. HK2 capture antibodies were dispensed on the P-Si chip and PSA spiked serum samples were incubated on the chip for testing the level of cross-reaction. The developed platform has the advantages of low sample consumption, time efficiency and robustness. It took in total time of around 3 h from dispensing the capture antibody on the surface to the end of the immunoassay.

## 2. Experimental Section

### 2.1. P-Si Fabrication

The porous silicon fabrication is governed by various etching parameters such as HF concentration, current density, anodization time, illumination, orientation of crystal, silicon types, doping level, *etc.* [39]. The porous silicon fabrication procedure in this paper followed optimum antibody immobilization conditions described previously [48]. Briefly, silicon, 6–8 Ω∙cm resistivity (boron doped p-type), <100> orientation, was purchased from Addision Engineering (San Jose, CA, USA). The wafer was placed in an electrochemical-etching cell. The electrolyte solution consisted of 3.6% hydrofluoric acid and 90.7% dimethylformamide (Merck, Darmstad, Germany). The silicon was anodized for 70 min with backside illumination. Current density during anodization was 90 mA/m^2^, after which the silicon was washed in ethanol three times and diced into 3 mm × 3 mm pieces to fit a microtiter plate format (Corning Costar Corporation, Cambridge, MA, USA).

### 2.2. PDMS Well

**Figure 2:**
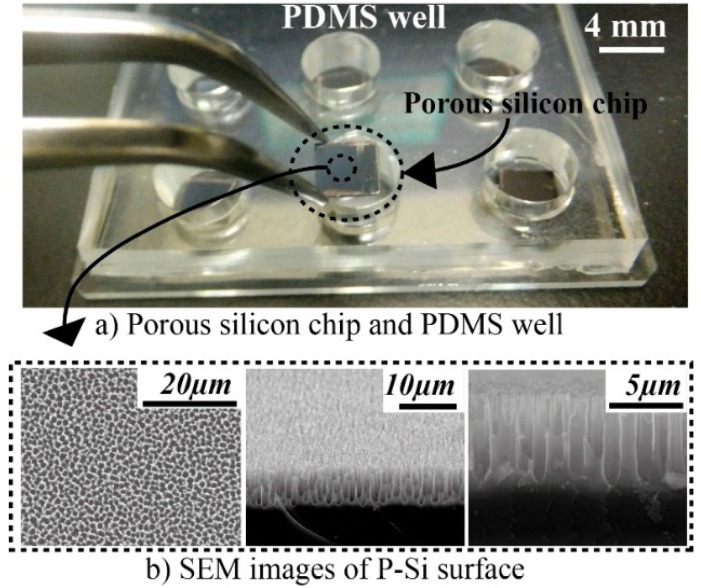
Porous silicon (P-Si) matrix used for microarrays. P-Si chips and PDMS wells (**a**) Capture antibody spotted P-Si chips were located in the wells to start the immunoassay. The scanning electron micrographs show a sequential zoom into a typical surface; (**b**) Macro-pores of micrometer size are clearly seen, combined with a micro- and nano-morphology (pore size around sub-µm to µm).

A 45 mm× 70 mm size and 5 mm thickness polydimethylpolysiloxane (PDMS) slab was prepared to well format as seen in Figure 2. The slab was fabricated with a Syglad 184 kit (Dow Corning Toray, Tokyo, Japan) that contains a PDMS monomer base and a curing elastomer reagent. The base and curing reagent were mixed in 10:1 weight ratio, and poured over a flat surface (*i.e.*, silicon or glass). The mixture was kept at 0.02 MPa for 30 min in a vacuum chamber to remove the trapped air bubbles from the liquid PDMS. After removing the bubbles, the mixture were baked at 70 °C for 1 h. Finally, 9 mm holes were punched on the PDMS slab to set up PDMS wells.

### 2.3. Proteins and Reagents

The monoclonal mouse antibody against hK2 (6B7, ab40749) was obtained by Abcam (Cambridge, UK). The polyclonal goat anti–hK2 antibody (PAB7226) and Alexa Fluor 488 labeled donkey anti-goat (ab150129) antibody were obtained at Abnova (Taipei City, Taiwan) and Abcam, respectively. Recombinant hK2 (ATGP2175) was obtained from ATGen (Pankyo, Korea) and prostate specific antigen (PSA) from human semen was obtained from Sigma-Aldrich (St. Louis, MO, USA).

### 2.4. Analytical Samples

Female single donor serum was purchased from BBI Solutions (S122–1, Cardiff, UK) and stored at −80 °C following the company’s recommendations. The serum was spiked with the recombinant hK2 in a titration series ranging from 100 fg/mL to 100 ng/mL.

### 2.5. Sandwich Assay

Monoclonal mouse capture antibody (6B7) for hK2 was dispensed onto the porous silicon chip, 3 × 3 mm size, using a piezoelectric dispenser (DW Scienion AB, Berlin, German) with a spot to spot distance of 150 µm. The antibodies were immobilized on the chip surface by physical adsorption. A drop volume was around 300 pL and a 13 × 13 (169 spot) array was spotted on the surface for each array. The microarray chips were placed in the PDMS well plates for the immunoassay. The porous silicon microarray immunoassay was evaluated using samples with hK2 spiked into both pure buffer (PBS) and female serum to obtain a final concentration ranging from 100 fg/mL to a few 100 ng/mL. The following steps were performed at room temperature:

1 After arraying the antibody, the chips were washed three times using 10 mM PBS to remove loosely bound antibodies.
2 The chips were incubated for 1 h in 100 µL blocking solution (5% (w/v) non-fat dry milk in PBS (Bio-Rad, Hercules, CA, USA)) to prevent non-specific binding and washed 3 times using 0.05% Tween 20 in 10 mM PBS.
3 Following blocking, the chips were incubated with 15 µL of spiked sample for 1 h, then washed (as above) and subsequently incubated with 15 µL of detection antibody (polyclonal goat anti-hK2).
4 After another washing step, 15 µL of AF488 labeled anti-goat polyclonal antibody was added onto the chips and incubated for 1 h.
5 Finally, the chips were washed three times and dried at room temperature.
6 The fluorescent spots images were observed using a eclipse TE2000-U fluorescence microscope (Nikon, Tokyo, Japan).
7 The captured images were analyzed by the open source image-processing tool Image J.

### 2.6. Mean Spots Intensities and Limits of Detection

The mean intensities of spots were measured and quantified by Image J among all microarray spot images, we selected nine spots (a 3 × 3 array) on each P-Si chip for quantification of the data since they fitted well in a single screen image. A total of 18 spots were chosen for data analysis since all experiments were performed on two independent chips. The spot intensities (S) were measured and quantified across the area. The local background (B) was collected in the same way and subtracted from the spot signals, generating mean spots intensities (S-B) as presented in the graphs. Mean spot intensity (S-B) can be defined as $\overset{n}{\underset{1}{\sum }}{\left(S-B\right)}_{n}/n$. The limit of detection (LOD) was followed 3-sigma (*σ*) defined as the lowest detectable signal from 3-sigma standard deviation above the mean spot intensities of negative control (N). The choice of LOD can be written as $>{\left(S-B\right)}_{N}+3\sigma$, when ${\left(S-B\right)}_{N}$ is mean spot intensity of negative control (N) and *σ* is standard deviation of negative control.

## 3. Results and Discussion

### 3.1. General Remarks

Surface bound immunoassays generally become more sensitive when the affinity or/and the density of the capture antibody increases [48]. The 3-D morphology of the micro/nano porous silicon surface layer offers a high antibody immobilization capacity and hence an increased antibody density per spot area [24,28]. Figure 2 shows macroporous layers with a characteristic size of sub micro −1 µm on the silicon surface recorded by Field Emission Scanning Electron Microscopy (FESEM).

### 3.2. hK2 Assay Performance against Density of Capture Antibody

**Figure 3:**
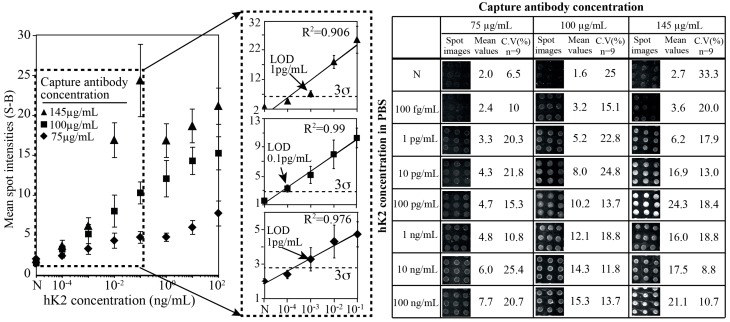
Titration series of hK2 in buffer (PBS) solution at three different concentrations of the capturing antibody 6B7 (75 µg/mL, 100 µg/mL and 145 µg/mL). The LOD was found to be 1 pg/mL when the capturing antibody was 75 µg/mL and was reduced to 100 fg/mL when the capturing antibody concentrations were 100 µg/mL. The LOD became 1 pg/mL again when concentration of the antibody was 145 µg/mL.

To optimize the P-Si hK2 immunoassay, we first evaluated the assay sensitivity by arraying the capture antibody, 6B7, at three different concentrations and performing the hK2 immunoassay in PBS buffer (10 mM, pH 7.2). Figure 3 shows a titration series of hK2 against three different capture antibody concentrations (75, 100 and 145 µg/mL).

Increasing concentrations of capture antibody yielded elevated fluorescent signals. However, the negative control (N) signal also increased at higher density of the antibody. The coefficient of variance (C.V) and mean spot intensities of arrays were shown in a right side of the figure. The fluorescent signal level of negative control (N) increased up to 70% (mean spot intensity is around 3) at a capture antibody concentration of 145 µg/mL compared to those at 75 and 100 µg/mL (mean spot intensity is around 2 at capture antibody 75 µg/mL and 1.95 at 100 µg/mL). In most cases, spot reproducibility within the chips shows good reliability (CVs~10%–20%) and also was in agreement with earlier results [33]. The linear regression of the mean spot intensities *versus* hK2 concentration corresponded to a coefficient of determination (R^2^) equal to 0.97, 0.99 and 0.906 for capture antibody concentrations of 75, 100 and 145 µg/mL, respectively.

It was hypothesized that the unspecific binding between the capture antibody and the secondary or the detection antibody increased at elevated capture antibody concentrations. To define the limit of detection, we chose 3-sigma (σ) method, which is the lowest signal detection at least three times above the standard deviation of the negative control (N). At a capture antibody concentration of 75 µg/mL the limit of detection (LOD) was found to be 1 pg/mL and at a capture antibody concentration of 100 µg/mL the LOD was improved by one order of magnitude to 100 fg/mL. We believe that the 100 fg/mL LOD reported for hK2 is competitive with the results of the DELFIA system for which a sub pg/mL LOD is reported [10,25].

A further increased density of capture antibody (145 µg/mL) did not yield an improved LOD as this also resulted in a significantly increased negative control level. The dynamic range was 10^5^ (10^−3^ to 10^2^ ng/mL) at a capture antibody concentration of 75 µg/mL and increased to 10^6^ (10^−4^ to 10^2^ ng/mL) when the capture antibody concentration was 100 µg/mL. The signal intensities were enhanced in proportion to the concentration of the capturing antibody, which followed the expectations of improved LOD with increased surface density of the capture antibody. However in case of 145 µg/mL capture antibody, the readout signal intensity drastically increased up to 100 pg/mL of hK2 level and it was saturated at higher concentrations (over 1 ng/mL hK2 level). The dynamic range therefore, defined a 10^2^ order (from 1 pg/mL to 100 pg/mL), which was reduced by two orders of magnitude.

### 3.3. hK2 Immunoassay in Female Human Serum

We evaluated the P-Si immunoassay platform subsequently in human serum since the final purpose of our platform development is to quantify the levels of hK2 in clinical samples. Initially, a high concentration of recombinant hK2 (over a few µg/mL) was spiked into a serum sample and the spiked samples were serially diluted up to 10^6^ times with serum to achieve total hK2 levels ranging from a hundred fg/mL to a hundred ng/mL. As a negative control we assayed unspiked serum.

**Figure 4:**
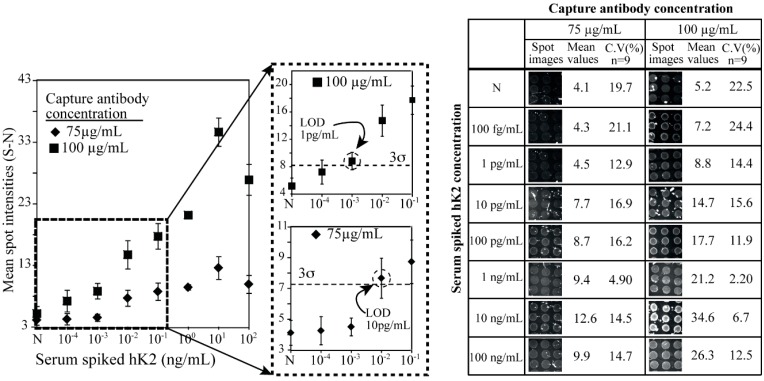
hK2-spiked human female serum analyzed with the sandwich microarray at two different capturing antibody concentrations (75 µg/mL and 100 µg/mL). The LOD was 10 pg/mL and 1 pg/mL when the concentrations of capture antibody were 75 µg/mL and 100 µg/mL, respectively. Increased assay sensitivity was observed with an elevated concentration of the capturing antibody (6B7). The negative signal also increased at the higher concentrations of the capture antibody.

We chose to spot capture antibody concentrations of 75 µg/mL and 100 µg/mL since they showed good assay readouts with a minimum negative (N) control (Figure 3). The corresponding titration series of hK2 in female serum was recorded for microarrays with the two different antibody concentrations (Figure 4). In the case of the 100 µg/mL antibody concentration, spot intensities could not be clearly distinguished against the negative controls (N) at an hK2 level of 100 fg/mL. The spot intensity of 1 pg/mL was however clearly distinguished (over 3 σ) from the negative control (N), and was defined as the LOD in the serum sample experiments. In the case of a capture antibody concentration of 75 µg/mL the signals were not distinguishable against the negative control until a level of 10 pg/mL of hK2 was reached (Figure 4).

From the sensitivity point of view, the serum spiked hK2 immunoassay at 75 µg/mL of capture antibody concentration showed the best assay performance. Total assay time is less than 3 h, which included 40 min for each blocking, sample incubation, primary antibody incubation and secondary incubation. All the microarray experiments had a coefficient of variation (CV) of less than 25%, indicating good spot reproducibility of the chips. The negative control signal (N) increased up to 100% as compared to the PBS buffer readout. This may be explained by cross-reaction of the antibody in the complex serum sample. Since serum contains various components such as nucleotides, peptides and proteins, one or some of these contents might have a cross-reaction with the antibodies. The intensity of the negative signal (N) is similar to the pg/mL level of hK2 in pure buffer condition and all intensities of serum sample are higher than those in PBS, which indicates a correlation between antibodies and serum.

**Figure 5:**
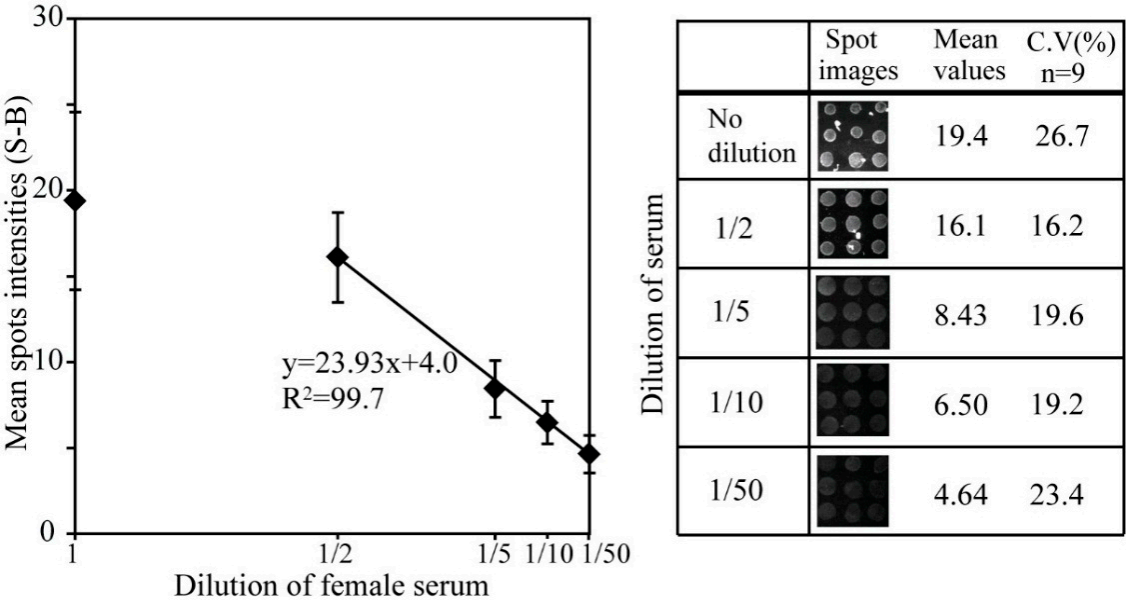
Immunoassay signals of the diluted serum samples. Serum samples were diluted down to 50 times and the samples were immunoassayed on microarray chips. The concentration of capture antibody was 100 µg/mL. Mean spot intensities and coefficients of variant are presented with spot images in the left panel.

Since the negative signal intensity (N) of the serum was similar to the intensity of 1 pg/mL of hK2 spiked PBS buffer sample, we further evaluated the platform through a serum PBS dilution series at four different concentrations (1/50, 1/10, 1/5, and 1/2). The microarray chips with 100 µg/mL of capture antibody were used for immunoassay of the four-dilution serum sample. Figure 5 shows readout signals of the microarray against the diluted serum samples. Dwindling signal was observed down to 50 times dilution. It could be caused by cross-reaction of several components in the serum to capture antibody and/or it also might be considered that intrinsic hK2 exists even in female serum that is extremely low levels not detected by a normal ELISA format.

### 3.4. Cross Reaction of hK2 Antibody against PSA

**Figure 6:**
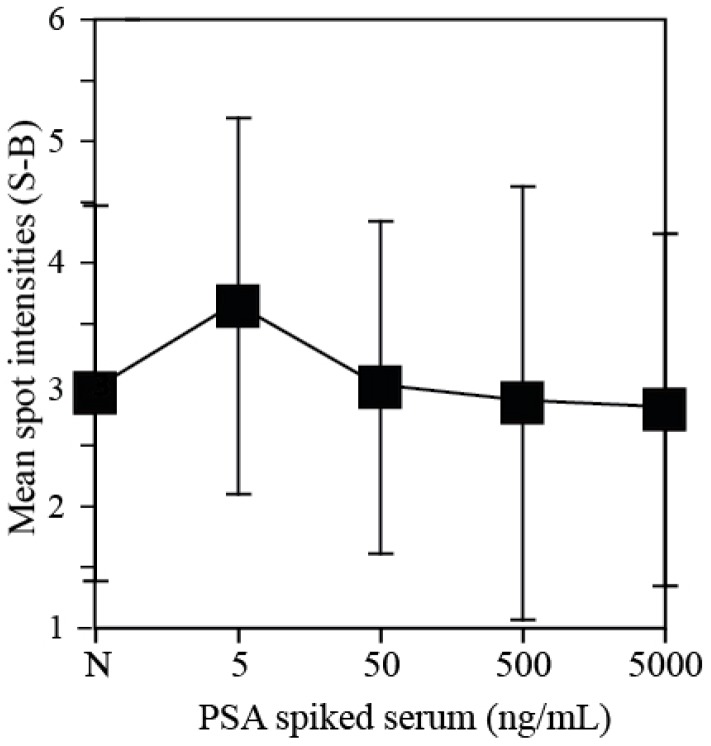
Cross-reaction tests of hK2 antibody spots against PSA spiked serum. Immunoassay signal of negative control (female serum sample) was compared with four PSA-spiked serum samples (5, 50 and 500 ng/mL and 5 µg/mL). HK2 capture antibody was spotted on P-Si chips at a concentration of 100 µg/mL.

Since human kallikrein 2 (hK2) has a high homology to prostate specific antigen (PSA) there is a critical need to carefully determine the hK2 assay design against PSA. We therefore performed serum- spiked PSA assays using the hK2 antibody microarrays to investigate the ratio of cross-reaction between PSA and hK2. We dispensed hK2 capture antibody with 100 µg/mL concentration on P-Si substrates and performed immunoassays of PSA-spiked serum at four different concentration (5, 50 and 500 ng/mL and 5 µg/mL). Figure 6 shows the readout microarray signals of PSA-spiked serum with negative control. All four signals of PSA-spiked serum are not significantly different from that of the negative control, from which it can be determined that there is no cross-reaction between hK2 capture antibody and PSA. The developed P-Si microarray platform therefore, is sufficient to quantify of hK2 abundance in serum without worrying about any cross-reaction with PSA.

## 4. Conclusions

This paper reports a simple and robust detection methodology for hK2 determination in serum samples. The large surface area of the tailored porous silicon used enables a fast and simple antibody immobilization by physisorption, and allows improved detection sensitivity and a broad dynamic range. The amount of antibody consumed on one chip was around 33.8 fmol (one spot is around 200 amol and the array has a 13 × 13 format), which is an exceptionally small amount compared to conventional ELISA formats.

The porous silicon microarray displayed a 100 fg/mL LOD and a dynamic range of 10^6^ (10^−4^ to 10^2^ ng/mL) under optimized capture antibody conditions. The developed assay platform also worked well in human serum and showed a LOD of 1 pg/mL and a dynamic range of 10^4^ (1 pg/mL to 10 ng/mL).

The simple physical-adsorption antibody immobilization avoided laborious and time-consuming chemical immobilization procedures, which shortens the overall assay time. It makes it possible to start the immunoassay a few minutes after the antibody immobilization process. The total assay time was about 3 h, including the antibody immobilization. Recent studies report that either the level of hK2 alone or in combination with total PSA (tPSA) or free PSA (fPSA) might improve prediction of PCa stage and risk of biochemical cancer recurrence after radical prostatectomy (RP) [49,50]. Since our assay can detect one or two orders of magnitude lower amounts of hK2 compared to the highly optimized ELISA system [10,25] it could contribute to future clinical practice by enabling more accurate diagnosis of prostate cancer patients. The usage of our microarray format can be extended to real clinical experiments for minimizing overdiagnosis of PCa, by evaluating hK2 levels in screened cohorts of men with PCa and higher PSA level, enrolled in first PSA screening rounds [49]. The simple method also has the merit of shortening the otal assay time and this makes it possible to analyze a large number of samples in time-saving way. Lastly, the large sample sizes have a significant positive influence on the power of statistical analysis.
